# Moderate- and High-Intensity Endurance Training Alleviate Diabetes-Induced Cardiac Dysfunction in Rats

**DOI:** 10.3390/nu15183950

**Published:** 2023-09-12

**Authors:** Sarah D’Haese, Maxim Verboven, Lize Evens, Dorien Deluyker, Ivo Lambrichts, BO Eijnde, Dominique Hansen, Virginie Bito

**Affiliations:** 1 UHasselt, Cardio & Organ Systems (COST), Biomedical Research Institute, Agoralaan, 3590 Diepenbeek, Belgium; sarah.dhaese@uhasselt.be (S.D.);; 2 Department of Internal Medicine, CARIM School for Cardiovascular Diseases, Maastricht University Medical Centre, Universiteitssingel 50, 6229 ER Maastricht, The Netherlands; 3 UHasselt, SMRC Sports Medical Research Center, Agoralaan, 3590 Diepenbeek, Belgium; 4 Division of Sport Science, Faculty of Medicine & Health Sciences, Stellenbosch University, Stellenbosch 7602, South Africa; 5 UHasselt, REVAL Rehabilitation Research Centre, Faculty of Rehabilitation Sciences, Agoralaan, 3590 Diepenbeek, Belgium; 6 Department of Cardiology, Heart Centre Hasselt, Jessa Hospital, Stadsomvaart 11, 3500 Hasselt, Belgium

**Keywords:** type 2 diabetes, Western diet, exercise training, diastolic dysfunction, adverse cardiac remodeling

## Abstract

Exercise training is an encouraging approach to treat cardiac dysfunction in type 2 diabetes (T2DM), but the impact of its intensity is not understood. We aim to investigate whether and, if so, how moderate-intensity training (MIT) and high-intensity interval training (HIIT) alleviate adverse cardiac remodeling and dysfunction in rats with T2DM. Male rats received standard chow (n = 10) or Western diet (WD) to induce T2DM. Hereafter, WD rats were subjected to a 12-week sedentary lifestyle (n = 8), running MIT (n = 7) or HIIT (n = 7). Insulin resistance and glucose tolerance were assessed during the oral glucose tolerance test. Plasma advanced glycation end-products (AGEs) were evaluated. Echocardiography and hemodynamic measurements evaluated cardiac function. Underlying cardiac mechanisms were investigated by histology, western blot and colorimetry. We found that MIT and HIIT lowered insulin resistance and blood glucose levels compared to sedentary WD rats. MIT decreased harmful plasma AGE levels. In the heart, MIT and HIIT lowered end-diastolic pressure, left ventricular wall thickness and interstitial collagen deposition. Cardiac citrate synthase activity, mitochondrial oxidative capacity marker, raised after both exercise training modalities. We conclude that MIT and HIIT are effective in alleviating diastolic dysfunction and pathological cardiac remodeling in T2DM, by lowering fibrosis and optimizing mitochondrial capacity.

## 1. Introduction

Type 2 diabetes (T2DM) remains a major health burden worldwide and its global prevalence keeps rising at an alarming rate [1]. Current estimations indicate that more than 463 million adults have T2DM, which is expected to reach 700 million by 2045 [2]. This observed trend is largely driven by modifiable lifestyle factors, including insufficient physical activity and an unhealthy diet [3]. An essential dietary determinant of T2DM is the increased consumption of refined sugars, in the form of glucose, fructose and sucrose, used extensively in the food industry [4,5]. Cohort studies have demonstrated an association between sugar-sweetened beverages and foods and the incidence of T2DM and diabetes-related comorbidities, including cardiovascular diseases (CVD) [6,7,8,9]. Indeed, individuals with T2DM are at increased risk of developing adverse cardiac remodeling and dysfunction, thereby increasing their susceptibility to developing heart failure (HF) [10,11]. The incidence of HF with T2DM is two- to five-fold higher in men and women, respectively, than in sex-matched individuals without T2DM [12].

Diabetic cardiomyopathy is a diabetes-induced heart muscle disease in which the myocardium has adverse structural and functional changes in the absence of coronary artery disease and valvular or congenital heart disorders [13]. In the early stage of T2DM, hyperglycemia, lipotoxicity and the presence of harmful, glycated proteins or so-called advanced glycation end-products (AGEs) drive the development of microvascular endothelial dysfunction as well the formation of reactive oxygen species (ROS) and inflammation [13,14]. These diabetes-related pathophysiological mechanisms cause structural remodeling of the myocardium, including enlargement of cardiomyocytes (i.e., cellular hypertrophy) and excessive deposition of interstitial collagen (i.e., interstitial fibrosis) by fibroblasts, leading to myocardial stiffening. These structural cardiac abnormalities manifest as, often asymptomatic, diastolic dysfunction, meaning an impaired left ventricular (LV) filling and relaxation echocardiographic parameters [15]. Clinically, T2DM patients with this so-called restrictive or heart failure with preserved ejection fraction (HFpEF) phenotype present with a normal ejection fraction, small LV cavity, thick LV walls and elevated filling pressures [16]. This phenotype is observed in approximately 75% of asymptomatic, normotensive individuals with well-controlled T2DM [17]. In a later stage of diabetic cardiomyopathy, although less prevalent, chronically elevated levels of myocardial ROS, AGEs as well as hypoxia (i.e., induced by microvascular dysfunction) might induce cardiomyocyte damage (e.g., loss of sarcomere structure) or cell death and further, extensive replacement fibrosis [13,14]. Clinically, these structural changes induce the development of systolic dysfunction, meaning the presence of LV dilation and impaired cardiac contractility, and patients present with a so-called dilated phenotype or heart failure with reduced ejection fraction (HFrEF) [16].

To date, a wide array of rodent models presenting structural and/or functional features of cardiac impairment in the context of T2DM is available [18,19]. As such, rodents can be genetically modified to become resistant to or deficient to the receptor for leptin, which is an adipocyte-originating hormone that regulates food intake and energy consumption. For example, db/db mice and Zucker diabetic fatty rats are well-known genetic models for T2DM. In addition, low concentrations of toxins that damage the insulin-producing β-cells, such as streptozotocin (STZ), can be used to induce diabetes. However, these genetic and chemically-induced models represent the human phenotype of T2DM insufficiently, showing non-clinically relevant mutations and STZ-related toxicities in other organs, respectively. Thus, none of these diabetic cardiomyopathy models perfectly mimics the human phenotype, forming one of the obstacles to improving patient care. Previously, our research group developed a T2DM rat model with cardiac comorbidities [20]. The major advantage of this model is that it is based on long-term intake of high-sugar, Western diet, being more clinically relevant regarding the societal situation. In addition, our animal model does not use chemical substances or genetical changes to represent the disease development and simulates the clinical phenotype, making this animal model and disease progression closer to what is seen in humans.

Current management of diabetic individuals with HF focusses on the control of systemic hyperglycemia and hyperinsulinemia, as well as on other adjunct strategies tackling hypertension and disturbed lipid profile [13]. The European Society for Cardiology (ESC) indeed recommends the combinational use of metformin, an antidiabetic agent, with sodium-glucose cotransporter 2 inhibitors as first-line treatment [21,22]. However, frail individuals with T2DM and HF often have several comorbidities and are subjected to multiple treatments, thereby being more susceptible to drug-related side effects [23,24]. Especially, a specific treatment for cardiac dysfunction associated with T2DM is missing. To cover this research gap, there is a need for effective, multifactorial approaches including non-pharmacological interventions, which are feasible in daily practice [21,23]. Endurance training is well-known to enhance cardiovascular and metabolic outcomes, including glycemic control, insulin sensitivity, lipid profile and cardiorespiratory fitness, thereby serving healthy and, likely, T2DM individuals [25]. To reduce cardiovascular risk, the American Heart Association and ESC recommend 150 min or more of moderate-to-vigorous intensity activity per week, over at least 3 days, for adults with T2DM [3,21]. Especially, the intensity of exercise training seems to be an important determinant in the treatment of T2DM individuals with cardiac comorbidities [25].

Until now, moderate-intensity training (MIT), which typically is at 40–69% of VO_2_max, was considered as the gold standard for exercise training recommendations in T2DM [21,23,26]. As such, a large prospective cohort study by Sluik et al. reported that physical activity, especially in moderately active persons, was associated with a lower risk of CVD and mortality risk in diabetic individuals [27]. However, recent studies have suggested that vigorous or higher-intensity exercise training might be equally effective or even more potent than the classical MIT approach to treat CVD in T2DM [25]. For this, high-intensity interval training (HIIT) is considered as a feasible strategy to reach a high-intensity level. HIIT is characterized by high-intensity bouts of short duration, typically at 70–95% of VO_2_max, alternated with short periods of active rest at low-to-moderate intensity [23,26]. Different reviews and meta-analyses report that small clinical trials including T2DM patients showed equal or superior effects of HIIT on body composition, glycaemic control, vascular complications and cardiorespiratory fitness as compared to MIT [28,29,30,31,32,33]. The combination of HIIT with resistance training and MIT with resistance training improved the intima-media thickness of the carotid artery in T2DM patients, whereas only HIIT had beneficial effects on peripheral arterial stiffness [30]. In addition, a randomized controlled trial demonstrated that 11 weeks of HIIT had similar improvements in body composition and showed better results in physical fitness compared to MIT in T2DM individuals [34]. Furthermore, a randomized controlled trial by Hwang et al. reported similar improvements in aerobic fitness in TD2M patients following MIT and HIIT [31]. Of note, this study also concluded that HIIT is a feasible, well-tolerated and safe approach for these individuals, indicating HIIT as a promising alternative for those unwilling to engage MIT. Given its shorter training duration, adherence to HIIT also has been shown to be high and comparable with MIT in patients attending cardiac rehabilitation [35]. Despite reducing cardiometabolic risk factors, the effect of HIIT specifically on cardiac function in T2DM is still unknown and more research is necessary.

Altogether, current management of diabetic heart failure focuses on one-for-all-type therapeutic treatments, while strategies especially targeting the diabetic heart are missing. Therefore, new approaches tackling cardiac dysfunction in T2DM, including lifestyle changes, should be investigated and, if effective, implemented in clinical care. HIIT has been proposed as a promising alternative for MIT in the treatment of general features in T2DM. Nevertheless, whether and how different intensities of exercise training can improve diabetes-associated structural and functional changes of the heart is unknown, which currently forms a specific knowledge gap in this research field. Therefore, we aim to investigate how MIT and/or HIIT could alleviate T2DM with adverse cardiac remodeling and dysfunction in rats. To address this goal, rats were fed a Western diet (WD) to induce T2DM with adverse cardiac remodeling and dysfunction for 18 weeks, followed by continued food intake in the presence of absence of MIT or HIIT treadmill running for 12 additional weeks. At the end of the study, metabolic and LV echocardiographic measurements were performed, followed by histological and colorimetric analysis of LV tissue. We hypothesize that MIT- and HIIT-induced cardiac benefits in WD-fed rats are mediated by decreased LV cardiac fibrosis and inflammation as well as improved mitochondrial oxidative capacity.

## 2. Materials and Method

### 2.1. Animal Experiments and Study Design

All animal experiments were conducted in accordance with the EU directive 2010/63/EU for animal testing and were approved by the Local Ethical Committee for Animal Experimentation (UHasselt, Diepenbeek, Belgium; ID201554). All animals were maintained in a temperature-controlled environment (21 °C, 60% humidity) on a 12 h light/dark cycle. Drinking water and food available was *ad libitum*. In total, 32 male Sprague–Dawley rats (Charles River Laboratories, L’Arbresle, France) were used in the study.

**Figure 1:**
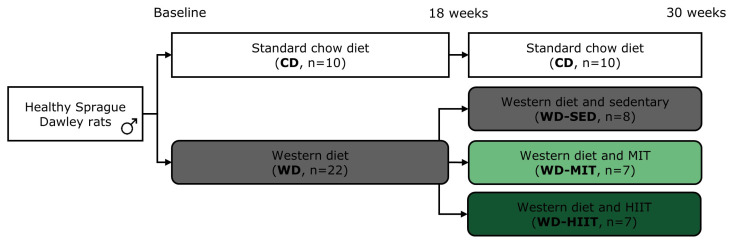
Experimental design of the study. Rats were maintained on a standard chow diet (CD) or Western diet (WD) for 18 weeks. After 18 weeks, WD rats were assigned to a sedentary lifestyle (WD-SED), moderate-intensity training (WD-MIT) or high-intensity interval training (WD-HIIT) for 12 additional weeks, while continuing the assigned diet. Evaluation of lipid profile, glucose tolerance and cardiac function was performed at baseline, 18 weeks and 30 weeks. Sample size is indicated as ‘n’.

Healthy rats, at 8 weeks of age and weighing 200–225 g, were randomly allocated into two groups (Figure 1). The first group received a standard chow diet (24% kcal% proteins, 18% kcal% fat, 58% kcal% carbohydrates from grains, no added sugars; Teklad Global Rodent Diet, ENVIGO, Horst, The Netherlands) (CD, n = 10) throughout the entire study. The second group was fed a high-sugar or so-called Western diet (15% kcal proteins, 16% kcal fat, 69% kcal carbohydrates, of which 48% kcal sugars from sweetened condensed milk and added sucrose) (n = 22) to induce T2DM with adverse cardiac remodeling and dysfunction [20,36]. After 18 weeks of diet (CD or WD), all rats continued their assigned diet while WD rats were also randomly subjected to a sedentary lifestyle (WD-SED, n = 8), MIT (WD-MIT, n = 7) or HIIT (WD-HIIT, n = 7) for 12 additional weeks. The total study duration was 30 weeks.

Body weight was measured every week. Blood sampling, echocardiographic measurements and an oral glucose tolerance test (OGTT) were performed at baseline, 18 weeks and 30 weeks after the start of the diet. Hemodynamic measurements were performed at the end of the protocol, just before the sacrifice. All procedures were performed under anesthesia (2% isoflurane supplemented with oxygen). At sacrifice, the animals were first injected with heparin (1000 u/kg, intraperitoneal (i.p.)) and euthanized with an overdose of sodium pentobarbital (200 mg/kg, i.p., Dolethal, Vetoquinol, Aartselaar, Belgium). The hearts and livers were harvested and weighted. Transversal sections of the heart were fixed in 4% paraformaldehyde overnight, transferred to 70% ethanol and embedded in paraffin for histological staining. Residual LV tissue was crushed into fine powder, immediately frozen in liquid nitrogen and stored at −80 °C for western blot and colorimetry analysis. A hind leg was removed for measurements of tibia bone length (TL) to normalize organ weights.

### 2.2. Exercise Protocol

Treadmill running (Expendable Treadmill Model 805, IITC Life Science, California, USA) was performed 5 days/week for 12 weeks, according to a training protocol previously described by Verboven et al. [37]. In brief, MIT consisted of continuous moderate-intensity running at 18 m/min for 1 h/day at 5° inclination. HIIT consisted of 10 bouts of 2 min high-intensity running (18 m/min at 30° inclination) separated by 1 min of active rest (12 m/min at 30° inclination). Exercise training intensity was assessed by measuring blood lactate levels directly after exercise training (Analox apparatus, Analis, Namur, Belgium). Levels > 4 mmol/L lactate were considered HIIT [37,38]. Training modalities were adjusted to result in equal energy expenditure between interventions by calculating the net caloric cost (kcal/min) using the following formulas [37]:

$${\mathrm{VO}}_{2}\max\;=\;S\;*\;0.2+\left(S\;*\;G\right)\;*\;0.9\;\left(S\;=\;\mathrm{speed}\;\left(m/\min\right),\;G\;=\;\mathrm{inclination}\right);$$

$$\mathrm{Net}\;\mathrm{caloric}\;\cos t\;\left(\mathrm{kcal}/\min\right)=\;{\mathrm{VO}}_{2}\max\;*\;3.5\;*\;\mathrm{body}\;\mathrm{mass}\;\left(\mathrm{kg}\right)/200.$$

### 2.3. Conventional Echocardiographic Measurements

Transthoracic echocardiography of the LV was performed in a supine position under 2% isoflurane anesthesia supplemented with oxygen in all animals (GE Vivid i, Vingmed Ultrasound) using a 10 MHz linear array transducer, as described previously [20]. Heart rate (HR) was monitored noninvasively while measurements were taken. A parasternal long-axis image and short-axis views at the mid-ventricular level were obtained at a temporal resolution of 200 frames per second. Conventional echocardiographic parameters including LV internal end-diastolic diameter (EDD); representing the diameter across the LV at the end of LV relaxation or filling, internal end-systolic diameter (ESD); representing the diameter across the LV at the end of LV contraction or ejection, posterior wall thicknesses (PWT) and anterior wall thicknesses (AWT) were obtained from the B-mode images at midpapillary level in the parasternal short-axis view. Fractional shortening (FS), representing the change in the diameter of the LV between the contracted and relaxed state, was calculated as $\left(\mathrm{EDD}\;-\;\mathrm{ESD}\right)/\mathrm{EDD}$, and expressed in %. End-systolic volumes (ESV), representing the amount of blood in the LV at the end of LV contraction or ejection, and end-diastolic volumes (EDV), representing the amount of blood in the LV at the end of LV relaxation or filling, were calculated by π∗DM2∗B/6, where DM indicates the systolic/diastolic diameter of the ventricle in mid-ventricular short-axis view and B is LV length on parasternal long-axis image. Subsequently, ejection fraction (EF), representing the amount of blood that is pumped out of the LV with each heartbeat, was calculated as $\left(\mathrm{EDV}–\mathrm{ESV}\right)/\mathrm{EDV}$, and expressed in %. To reduce bias, analysis of the echocardiographic data was first coded by an independent researcher and analysis was performed blinded.

### 2.4. Hemodynamic Measurements

LV pressure measurements (i.e., end-diastolic pressure (EDP), end-systolic pressure (ESP) and time constant for isovolumetric relaxation (Tau)) were performed at sacrifice, as described previously [20]. In brief, hemodynamic parameters were measured invasively with a pre-calibrated SPR-320 MikroTip high-fidelity pressure catheter (Millar Inc., The Hague, The Netherlands) advanced into the LV via the right carotid artery. The pressure catheter was connected to a quad-bridge amplifier and PowerLab 26T module (AD Instruments, Oxford, UK). Data were transferred to the data to LabChart v7.3.7 software (AD Instruments) for offline analysis.

### 2.5. Oral Glucose Tolerance Test and Insulin Resistance Assessment

Glucose tolerance was assessed at baseline, 18 weeks and 30 weeks after the start of the diet, with a 1 h OGTT as described by Stevens et al. [39]. After 16 h overnight fasting, glucose (2 g/kg) was administered via oral gavage. Before and 15, 30 and 60 min after administration, blood glucose concentration was determined from capillary tail blood collection (Analox GM7, Analis SA, Namur, Belgium). At baseline and after 60 min, plasma insulin concentrations were measured by electrochemiluminescence (Meso Scale, Gaithersburg, MD) [20]. The homeostasis model assessment of insulin resistance (HOMA-IR) was used to determine insulin resistance [40]. HOMA-IR was calculated from fasting glucose and insulin values using the following formula: $\mathrm{HOMA}-\mathrm{IR}\;=\left(\mathrm{fasting}\;\mathrm{insulin}\;\mathrm{levels}\;\left[µ\mathrm{IU}/\mathrm{mL}]\;*\;\mathrm{fasting}\;\mathrm{glucose}\;\mathrm{levels}\;[\mathrm{mmol}/L\right]\right)/22.5$.

### 2.6. Lipid Profile Assessment

Fasted plasma triglycerides and total cholesterol were determined in the Ziekenhuis Oost–Limburg (Genk, Belgium) using Roche/Hitachi cobas c systems (Rotkreuz, Switzerland). Quantification of fasted plasma free fatty acids (FFA) was assessed using an FFA quantification assay kit (Abcam, ab65341, Cambridge, UK) according to the manufacturer’s instructions.

### 2.7. Plasma Advance Glycation End-Products Determination

Plasma AGEs were determined with an OxiSelect Advanced Glycation End Product Competitive ELISA kit (Bio-Connect, Huissen, The Netherlands). In short, samples were added to the AGEs conjugate pre-absorbed ELISA plate. Subsequently, wells were incubated with a diluted anti-AGEs antibody and a horseradish peroxidase-conjugated secondary antibody was added.

### 2.8. Interstitial Fibrosis Measurement

Midventricular transversal sections of paraffin-embedded LV tissue (7 μm) were stained using the Sirius Red/Fast Green kit (Chondrex Inc., Redmons, WA, USA), according to the manufacturer’s instructions. After staining, sections were dehydrated in increasing concentrations of ethanol and mounted with a DPX mounting medium. Interstitial fibrosis was assessed in four randomly chosen 20× zoomed-in images using the Mirax Slide Scanner System (Carl Zeiss MicroImaging, Zaventem, Belgium). The area of collagen deposition was outlined, quantified using an automated image analysis program (Carl Zeiss, AxoVision 4.6, Zaventem, Belgium) and normalized to total surface area in %.

### 2.9. Western Blot on LV Tissues

Protein concentrations were determined by the BCA protein assay kit (Thermo Fisher, Erembodegem, Belgium). Western blot was performed as previously described [41]. In brief, equal amounts of proteins (15 µg) were separated on a 12% SDS-PAGE gel with a mini protean 3 electrophoresis system (Bio-rad Laboratories, Temse, Belgium), transferred to a polyvinylidene fluoride membrane and subsequently, blocked for 2 h with 5% milk in Tris-buffered solution containing 0.1% Tween-20 (TBS-T) followed by incubation overnight at 4 °C in the presence of a tumor necrosis factor α (TNF-α) antibody (1/1000, goat polyclonal IgG, Santa Cruz, N-19, Heidelberg, Germany) or lysyl oxidase (LOX) antibody (1/1000, rabbit polyclonal IgG, Abcam, ab31238, Cambridge, UK). Horseradish peroxidase-conjugated secondary antibodies (DAKO, Belgium) at a dilution of 1/2000 were used. Both primary and secondary antibodies were diluted in 5% milk-TBS-T. Visualization was performed with the enhanced chemiluminescence (ECL) technique using the Pierce ECL Plus western Blotting Substrate (Thermo Fisher, Erembodegem, Belgium). Data were normalized to β-actin protein levels.

### 2.10. Citrate Synthase Activity from LV Homogenates

Citrate synthase activity was determined using a citrate synthase assay kit (CS0720; Sigma-Aldrich, St. Louis, MO, USA) according to the manufacturer’s instructions [37].

### 2.11. Statistical Analysis

Statistical analyses were performed using Graphpad Prism (Graphpad Software, version 9.3.0, San Diego, CA, USA). Power analysis were performed using G*Power (G*Power software, version 3.1.9.4, Dusseldorf, Germany) to estimate the smallest sample size needed for the study to reach 80% statistical power. Outliers were detected using the ROUT test method with a maximum desired False Discovery Rate of 1%. The normal distribution of data was evaluated with the Shapiro–Wilk test. Normally distributed data were subjected to either an unpaired t-test or a one-way ANOVA followed by Tukey’s multiple comparisons test. For data not normally distributed, either a Mann-Whitney U test or a Kruskal Wallis test followed by Dunn’s multiple comparison test was applied. All data are expressed as mean ± standard error of the mean (SEM). The sample size is indicated as ‘n’. A value of *p* < 0.05 was considered statistically significant.

## 3. Results

### 3.1. Western Diet Intake Induces Obesity, Type II Diabetes and Cardiac Hypertrophy

**Figure 2:**
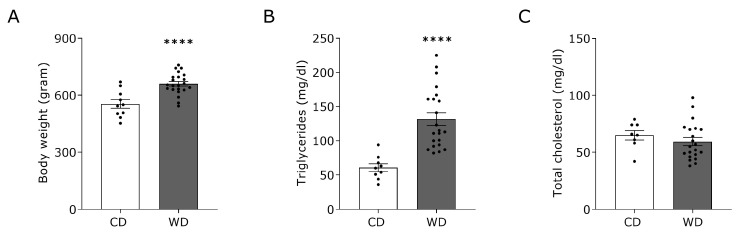
Body weight and blood lipid profile of rats receiving a Western diet for 18 weeks. Metabolic characteristics of rats receiving CD (n = 10) or WD (n = 22) for 18 weeks. (**A**) Body weight. (**B**) Fasting plasma triglyceride levels. (**C**) Fasting plasma total cholesterol levels. Data represent mean ± SEM. **** denotes *p* < 0.0001 vs. CD.

To confirm WD-induced T2DM with an adverse cardiac structural and functional remodeling in the rat model, measurements of blood lipid profile, glucose tolerance and echocardiography were conducted. As expected, 18 weeks of WD promoted a significant body weight gain compared to CD-fed rats (Figure 2A). In addition, fasting plasma triglyceride levels were significantly increased (Figure 2B) whereas total cholesterol levels were not altered with WD (Figure 2C).

**Figure 3:**
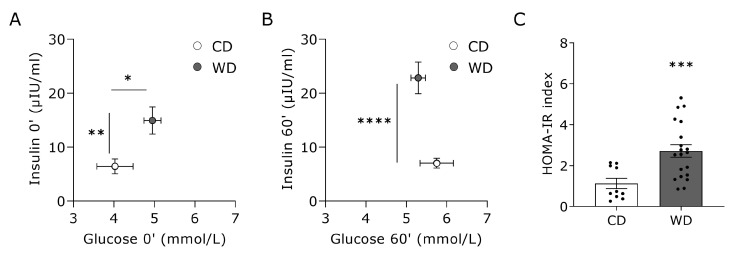
Glucose tolerance and insulin sensitivity of rats receiving a Western diet for 18 weeks. Glucose tolerance was assessed in rats receiving CD (n = 10) or WD (n = 22) for 18 weeks. Insulin levels as a function of glucose levels were obtained (**A**) at fasting or (**B**) 60′ post-glucose administration during an oral glucose tolerance test. (**C**) HOMA-IR index in both groups. Data represent mean ± SEM. * denotes *p* < 0.05, ** denotes *p* < 0.01, *** denotes *p* < 0.001 and **** denotes *p* < 0.0001 vs. CD.

Fasting plasma glucose and insulin levels were significantly elevated after 18 weeks of WD (Figure 3A). Moreover, rats undergoing 18 weeks of WD displayed significantly higher insulin levels 60 min post-glucose administration, indicating an impaired glucose tolerance (Figure 3B). The calculated HOMA-IR value, an indicator for insulin resistance, was significantly increased after WD intake compared to CD (Figure 3C).

In Vivo LV cardiac function and structure were evaluated with echocardiographic measurements. Cardiac parameters at week 18 are shown in Table 1. WD intake caused LV cardiac alterations in rats. Both AWT and PWT, markers for cardiac hypertrophy, were significantly increased in the hearts of rats undergoing WD. However, EF and FS, measures for global cardiac function, remained preserved after 18 weeks of WD intake. No significant changes were observed in cardiac diameters and volumes, stroke volume (SV) and cardiac output (CO) between the groups.

### 3.2. Exercise Training Reduces Body Weight Gain and Improves Blood Lipid Profile in Western Diet Fed Rats

**Figure 4:**
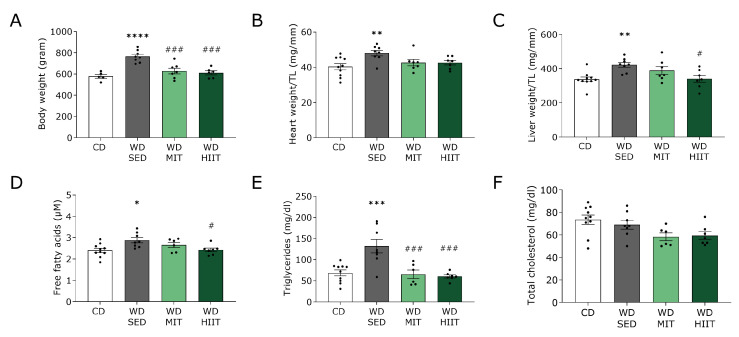
Effect of exercise training on body weight, liver weight and blood lipid profile of 30-week Western diet fed rats. Biometric and metabolic characteristics of rats receiving a CD (n = 10) or either kept WD-SED (n = 8), undergoing WD-MIT (n = 7), or WD-HIIT (n = 7) for 12 weeks. (**A**) Body weight. (**B**) Heart weight to tibia length (TL) ratio. (**C**) Liver weight to TL ratio. Fasting plasma (**D**) free fatty acid, (**E**) triglycerides and (**F**) total cholesterol levels. Data represent mean ± SEM. * denotes *p* < 0.05, ** denotes *p* < 0.01, *** denotes *p* < 0.001 and **** denotes *p* < 0.0001 vs. CD. ^#^ denotes *p* < 0.05 and ^###^ denotes *p* < 0.001 vs. WD-SED.

Biometric and metabolic characteristics of WD-fed rats after an additional 12-week of WD intake with or without exercise training are shown in Figure 4. Thirty weeks of WD combined with a sedentary lifestyle led to a significantly increased body weight compared to CD (Figure 4A). Both MIT and HIIT significantly reduced the body weight gain in WD-fed rats to the levels of CD. WD-SED-fed rats displayed significantly heavier hearts, demonstrated by an increase in heart weight/tibia length ratio, compared to CD-fed rats (Figure 4B). Although not significant, the increased heart weight to tibia length ratio seen in WD-SED was restored to the levels measured in CD with both exercise training modalities (CD; 40.35 ± 1.77, WD-SED; 48.05 ± 1.54, WD-MIT; 42.67 ± 1.85, WD-HIIT; 42.61 ± 1.30, Figure 4B).

In addition, WD-SED-fed rats displayed significantly heavier livers, accompanied by increased plasma FFA and triglyceride levels when compared to CD-fed rats (Figure 4C–E). Both exercise training protocols significantly lowered triglyceride levels in WD-fed rats, while only HIIT significantly decreased liver weight and circulating FFA levels, to CD levels. Plasma total cholesterol levels were comparable in all groups (Figure 4F).

### 3.3. Exercise Training Ameliorates Glucose Tolerance and Insulin Sensitivity in Western Diet Fed Rats

**Figure 5:**
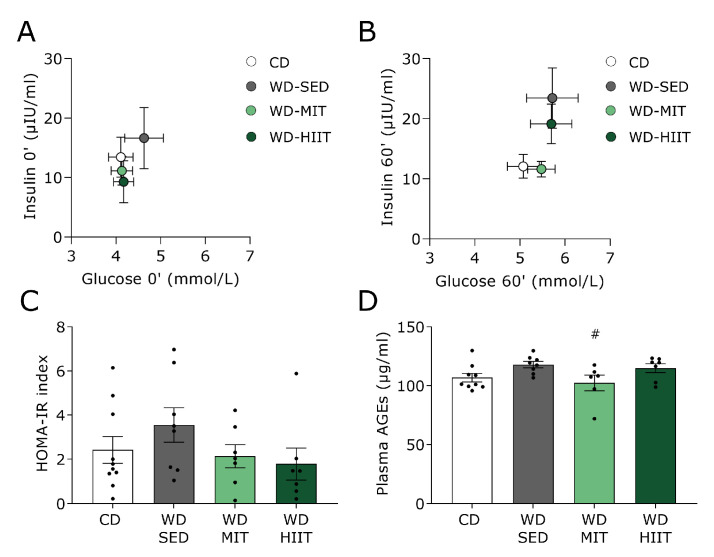
Effect of exercise training on glucose tolerance, insulin sensitivity and plasma advanced glycation end-products of 30-week Western diet fed rats. Glucose tolerance was assessed in rats receiving a CD (n = 10) or either kept WD-SED (n = 8), undergoing WD-MIT (n = 7), or WD-HIIT (n = 7) for 12 weeks. Insulin levels as a function of glucose levels were obtained (**A**) at fasting or (**B**) 60′ post-glucose administration during an oral glucose tolerance test. (**C**) HOMA-IR index. (**D**) Total plasma advanced glycation end-products (AGEs). Data represent mean ± SEM. ^#^ denotes *p* < 0.05 vs. WD-SED.

To investigate the effect of MIT and HIIT on glucose tolerance and insulin sensitivity, an OGTT was performed in WD-fed rats after 30 weeks of diet (Figure 5). WD-SED animals tended to display increased fasting plasma glucose and insulin levels compared to CD-fed rats (Figure 5A). Both exercise protocols slightly decreased fasting glucose and insulin levels in rats undergoing WD compared to CD-fed rats. In addition, rats undergoing the WD and having a sedentary lifestyle for 30 weeks displayed a trend toward increased insulin levels 60 min post-glucose administration compared to CD-fed rats (CD; 12.06 ± 1.98, WD-SED; 23.44 ± 5.03, *p* = 0.06, Figure 5B). MIT but not HIIT training tended to reduce the insulin levels 60 min post-glucose administration, compared to WD-fed rats kept sedentary (WD-SED; 23.44 ± 5.03, WD-MIT; 11.61 ± 1.31, *p* = 0.08, Figure 5B). Although not significant, this trend was also observed in the HOMA-IR values (CD, 2.42 ± 0.61; WD-SED, 3.55 ± 0.78; WD-MIT, 2.14 ± 0.53; WD-HIIT, 1.79 ± 0.72, Figure 5C). Despite being not significant, plasma AGE levels tended to be elevated in WD-SED rats compared to CD (CD; 106.80 ± 3.62, WD-SED; 117.90 ± 2.66, Figure 5D). Interestingly, MIT but not HIIT significantly lowered the levels of plasma AGEs compared to those of WD-SED animals to the CD levels.

### 3.4. Exercise Training Reverses LV Hypertrophy and Restores LV Function in Western Diet Fed Rats

Echocardiography measurements were performed at the end of the study to assess LV cardiac function and structure (Table 2, upper part). After 30 weeks of WD, WD-SED rats displayed a significantly increased AWT and PWT, which is a hallmark of a hypertrophic LV wall. Both MIT and HIIT significantly decreased PWT to CD levels. The increased ESV and ESD after 30 weeks of WD intake were not significantly improved by exercise training. Furthermore, WD-SED rats had a significantly reduced EF and FS, indicating impaired systolic dysfunction, compared to CD-fed rats. 12 weeks of MIT, but not HIIT, significantly restored EF and FS. Diastolic cardiac diameter and volume, SV and CO remained comparable in all groups.

The alterations in LV cardiac morphology were associated with changes in LV cardiac function, evaluated with hemodynamic measurements after 30 weeks of WD (Table 2, lower part). WD-SED rats had significantly increased EDP and ESP, indicators of cardiac diastolic and systolic function, respectively, compared with CD-fed rats. Both exercise training modalities significantly restored EDP, but not ESP, to CD levels. Even if the time constant for isovolumetric relaxation, Tau, was not statistically different from all groups, likely due to a large variability in the WD-SED group, values in the exercise training groups were closer to CD rather than WD-SED.

### 3.5. Exercise Training Reduces LV Fibrosis in Western Diet Fed Rats

**Figure 6:**
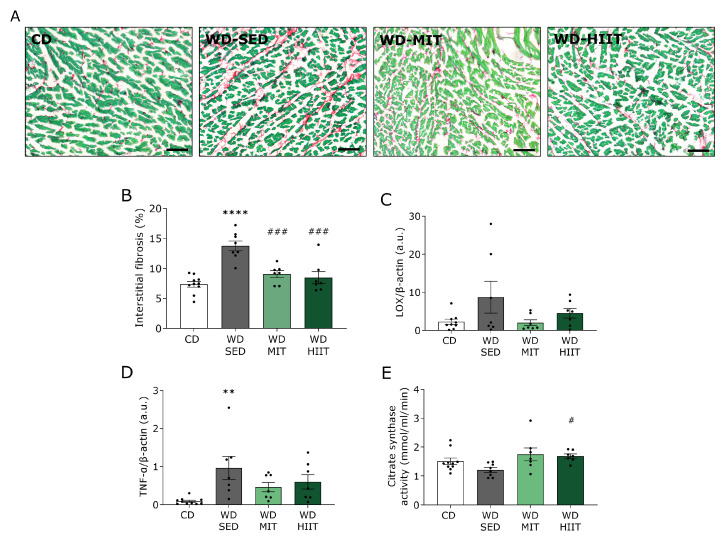
Effect of exercise training on left ventricular interstitial fibrosis, inflammation and mitochondrial mass after 30 weeks of Western diet. Markers for interstitial fibrosis, inflammation and mitochondrial mass assessed in the left ventricle of rats receiving a CD (n = 10) or either kept WD-SED (n = 8), undergoing WD-MIT (n = 7), or WD-HIIT (n = 7) for 12 weeks. (**A**) Representative 20x zoomed-in pictures of interstitial collagen obtained with Sirius Red/Fast Green in the left ventricle. Fibrotic tissue is stained red while cardiac cells are stained green. Scale bar represents 100 µm. (**B**) Percentage of total interstitial collagen deposition per surface area in the left ventricle. (**C**) Lysyl oxidase (LOX) protein expression normalized to β-actin. (**D**) Tumor necrosis factor α (TNF-α) protein expression normalized to β-actin. (**E**) Citrate synthase activity in left ventricular homogenates. Data represent mean ± SEM. ** denotes *p* < 0.01 and **** denotes *p* < 0.0001 vs. CD. ^#^ denotes *p* < 0.05 and ^###^ denotes *p* < 0.001 vs. WD-SED.

Representative pictures of interstitial collagen obtained with Sirius Red/Fast Green staining in LV sections of the four groups are shown in Figure 6A. Total interstitial collagen was significantly increased in WD-SED rats compared with CD rats after 30 weeks of diet (Figure 6B). Both MIT and HIIT significantly normalized the interstitial collagen deposition to CD levels. To further identify the involvement of collagen cross-linking, LOX levels were measured in LV cardiac homogenates. Despite not being significant, WD-SED animals tended to have increased LOX levels compared to CD-fed rats (CD; 2.24 ± 0.70, WD-SED; 8.71 ± 4.17, Figure 6C). Although not significant, predominantly MIT tended to reduce LOX protein levels compared to WD-SED (WD-MIT; 1.99 ± 0.77, Figure 6C).

Finally, we evaluated the effect of MIT and HIIT on LV inflammation and oxidative status. Protein levels of TNF-α, a pro-inflammatory cytokine, were significantly elevated after 30 weeks of WD intake and a sedentary lifestyle (Figure 6D). MIT tended to decrease the levels of TNF-α (WD-SED; 0.96 ± 0.30, WD-MIT; 0.46 ± 0.12, Figure 6D). In addition, citrate synthase activity, a marker for aerobic capacity and mitochondrial mass, significantly raised after HIIT and tended to improve after MIT (WD-SED; 1.51 ± 0.11, WD-MIT; 1.21 ± 0.08, WD-HIIT; 1.69 ± 0.08, *p* = 0.08 WD-SED vs. WD-MIT, Figure 6E).

## 4. Discussion

Endurance exercise training is currently recommended in the management of CVD patients with T2DM and/or obesity [21,23]. However, the optimal composition of exercise training, including duration, frequency and intensity, to tackle cardiac features in T2DM remains unclear. This study is the first to address the curative impact of exercise intensity on cardiac remodeling and dysfunction in a translatable, WD-induced T2DM rat model. We have shown that 12 weeks of either MIT or HIIT halt the development of diabetes and reverse LV diastolic dysfunction and remodeling. The beneficial cardiac effects of endurance exercise training are proposed to be mediated by limiting myocardial fibrosis, reducing inflammation and ameliorating mitochondrial oxidative capacity.

### 4.1. Diet-Induced T2DM Rat Model with Cardiac Structural and Functional Remodeling

Lately, the field has made major progress in the development and better characterization of preclinical models of T2DM with CVD [18,19]. One of the most extensively used models to study prediabetes and/or obesity in laboratory animals involves the consumption of a high-fat diet (HFD), lacking a refined sugar component [42]. These HFD-induced models often reproduce common hallmarks of the human phenotype, including weight gain, disturbed lipid profile and decreased insulin sensitivity. Indeed, obesity and its excessive accumulation of adipose tissue are causally linked to T2DM development [43]. Nevertheless, inconsistencies regarding the potential of HFD to induce LV cardiac dysfunction remain, as different studies have failed to identify cardiac complications whereas others have shown structural remodeling and diastolic dysfunction [44,45,46,47]. These conflicting data presumably arise from differences in rodent strains, timing and duration of the dietary interventions and diet composition, also making comparison difficult and outcome variability higher [48]. In addition, preclinical studies on the HFD regime mainly fail to account for later systolic dysfunction or reduced EF [18,49]. To ensure development of a diabetic state and LV cardiac dysfunction, an injection with low-dose STZ, a cytotoxin that damages pancreatic β-cells, is often added to the HFD regime [50,51]. However, this chemical-induced model may also present toxicities to other organs related to STZ, lowering its translational relevance [52].

Interestingly, data demonstrate that excessive sugar intake rather than an increased fat intake is associated with the current T2DM pandemic [53,54]. Although not yet extensively studied, dietary interventions high in monosaccharide fructose caused insulin resistance, unchanged or mildly elevated blood glucose, dyslipidemia and notable diastolic function in rodents [55,56,57]. Velagic et al. demonstrated that cardiac complications were more advanced in STZ-induced diabetic rats on a high-sugar HFD compared with those on a moderate-sugar HFD for 8 weeks, indicating the crucial role of sugars in diet composition in preclinical models for diabetic heart diseases [58]. Furthermore, elevated myocardial fructose has been shown to be an early cardiac response to diabetes, preceding clinically-detected diastolic dysfunction [59,60]. In our study, animals were fed a diet high in sucrose, a disaccharide of fructose and glucose, or so-called WD, in the absence of STZ or genetically changed make-up. We found that 18 weeks of WD induced a significantly increased body weight, impaired glucose tolerance, insulin resistance and disturbed lipid profile in rats, hallmarks also seen in patients. Besides T2DM development, rats demonstrated LV hypertrophy whereas EF remained preserved. Our findings are in line with the study of Verboven et al. in which 18 weeks of WD led to T2DM and diastolic dysfunction, characterized by an increased EDP or preload [20].

After 30 weeks of WD, rats displayed persistent signs of insulin resistance together with a significantly altered metabolic profile and heavier liver. Interestingly, insulin resistance is known to induce FFA release from adipose tissue, which can be taken up by liver cells and stored as triglycerides after re-assembling [61]. Whether hepatic steatosis is present in our model requires more investigation but is often observed in T2DM patients [62]. Besides metabolic changes, WD rats displayed cardiac structural and functional remodeling characterized by an increase in heart weight, higher EDP, LV wall thickness and increased interstitial myocardial fibrosis, indicating pathological hypertrophy. Indeed, it is known that high glucose levels stimulate cardiac fibroblasts to transform into myofibroblasts, which is also associated with cardiomyocyte switching to a fibrotic phenotype, further triggering cardiac fibrosis and inflammation [63]. On top of the observed diastolic dysfunction, 30 weeks of WD resulted in increased end-systolic volume and pressure associated with a reduced EF, all indicating an increased afterload, which is typical to chronic hypertension also seen in T2DM patients [64]. Taken together, our rat model developed a mild T2DM phenotype with adverse cardiac remodeling and diastolic dysfunction (i.e., at 18 weeks) and, later systolic dysfunction (i.e., at 30 weeks) thereby meeting clinical reality. Consistent with our findings, Maurya et al. recently demonstrated that long-term intake of a moderate-sugar (35% kcal carbohydrates of which 18.9% kcal sucrose) but also high-fat (45% kcal) diet leads to glucose and insulin intolerance, together with impaired diastolic function (i.e., at 20 weeks) and, later systolic dysfunction (i.e., at 24 weeks) in C57BL/6 male mice [65]. To the best of our knowledge, we are the first to investigate the long-term impact of a high-sucrose diet (69% kcal carbohydrates, of which 48% kcal sugars from sweetened condensed milk and added sucrose) on the heart of rats in the context of T2DM. Particularly, T2DM and its cardiovascular complications develop in a chronic manner most often in middle-aged or older adults [66]. Contrary to the clinical scenario, the majority of studies on diet-induced diabetic cardiomyopathy models last a few weeks and/or employ young rodents [48]. The little evidence regarding the chronic impact of diet on cardiometabolic outcomes emphasizes the need for preclinical investigation at different life stages.

### 4.2. MIT and HIIT as Potential Strategies to Reverse Cardiac Impairment in T2DM

A recent systematic review stated that exercise intervention, representing aerobic, anaerobic and resistance training, improves cardiometabolic health in obese individuals [67]. Additionally, continuous moderate-to-high-intensity exercise training has been demonstrated to be associated with a reduction in HbA1c and HOMA-IR values in T2DM patients [68,69,70]. Despite knowledge of cardiometabolic health in T2DM following endurance exercise training expanded in the last few years, it remains to be established whether these improvements are intensity-dependent [71]. Here, we show that even though very different in design, MIT and HIIT are equally effective in attenuating body weight gain and partially restoring altered glucose and lipid metabolism induced by WD. Rats undergoing HIIT also had lower plasma FFA levels and liver weights, in line with the findings of Lund et al. in HFD-fed mice undergoing HIIT for 10 weeks [72]. In accordance with our findings, prolonged MIT [73] and HIIT [74] also reduced liver fat content in T2DM individuals. It should be noted that intense endurance training has also been demonstrated to induce liver damage (e.g., increased lactate dehydrogenase) in marathon runners [75,76]. Despite being of special interest, we did not investigate liver samples in the current study, which we acknowledge to be a limitation. In addition, both exercise training modalities tended to decline HOMA-IR values in the present study, but only MIT inhibited a rise in insulin 60 min after glucose administration during OGTT. In line with this finding, Kar et al. showed a trend toward improvement in insulin sensitivity, measured during an intraperitoneal insulin tolerance test, following 20-week MIT in HFD-induced diabetic mice [77]. Overall, our findings indicate that HIIT is more capable in managing systemic dyslipidemia whereas MIT is better in reducing the degree of insulin resistance. Partially in line with our results, Hafstad et al. demonstrated improved liver triglycerides, FFA content and liver weight after 8-week MIT and HIIT in diet-induced obese mice [78]. The difference in dietary source, HFD and thus higher exogenous lipid content, might explain the effectiveness of both exercise training modalities on the lipid profile. Although the authors observed a normalized glucose tolerance after HIIT but not MIT, no insulin measurements have been reported which is a limitation of their study. Indeed, clinical diagnosis of prediabetes or T2DM is mainly based on HbA1c or fasting plasma glucose levels, as insulin resistance testing is practically complex [79]. However, as hyperglycemia and insulin resistance are the main hallmarks of diabetic cardiomyopathy, measurement of both metabolites is recommended to fully characterize the phenotype [80].

In T2DM adults, an overall healthy lifestyle including a healthy diet combined with regular exercise training is associated with a lower risk of incident CVD and mortality [81]. Here, we showed that MIT and HIIT are equally effective in improving diastolic function in WD-fed rats. Our results are consistent with others, showing improved hemodynamic measurements for diastole (i.e., EDP, peak rate of pressure decline, Tau) following MIT and HIIT in HFD-induced diabetic animal models [77,78,82,83]. Three studies have previously indicated that HIIT potentially induced physiological cardiac hypertrophy or a so-called athlete’s heart, assessed as increased heart weight and size and improved cardiac function, in HFD-induced T2DM mice [72,78,83]. Additionally, three studies on HFD-induced T2DM mice reported no changes in heart weight [77] or PWT [84,85] following MIT, suggesting the absence of physiological hypertrophic effects. Here, we are the first to show a decreased heart weight and PWT following both MIT and HIIT in high-sucrose diet-induced diabetic cardiomyopathy, potentially indicating the therapeutic nature of endurance exercise training against pathological cardiac hypertrophy. Unlike our high-sucrose diet study, the previous studies did not observe pathological hypertrophy following the diet, potentially explaining the different effects of endurance exercise training. Besides improving diastolic dysfunction, MIT also normalized systolic function by significantly increasing EF and FS in the WD-fed rats. In line with our findings, an increased EF and FS were observed following 8 weeks and 12 weeks of MIT in HFD and STZ-induced rats and mice, respectively [84,85]. Furthermore, 8 weeks of HIIT has been demonstrated to only improve diastolic function (i.e., the ratio of peak mitral flow velocity versus peak mitral annular velocity (e/e’)), whereas systolic function (i.e., FS and EF) remained unchanged in HFD-induced diabetic mice [72]. On the contrary, Khakdan et al. reported that 8 weeks of HIIT improved EF and FS in high-fructose diet and HFD-induced diabetic rats [86]. This was confirmed in the clinic as 12 weeks of both MIT and HIIT improved early diastolic tissue velocity (peak septal mitral annulus velocity in early filling phase (e’)) while only HIIT also ameliorated systolic parameters (i.e., peak systolic annular velocity, global strain) in individuals with T2DM [87]. A small clinical trial of Cassidy et al. also showed physiological cardiac hypertrophy along with improvements in early diastolic filling rates and EF in T2DM patients following 12 weeks of HIIT, demonstrating its therapeutic effects on cardiac relaxation and contraction [74]. Lastly, we could not detect changes in CO after MIT and HIIT in WD-fed rats. This was comparable with a study by Van Ryckeghem et al., demonstrating that MIT and HIIT ameliorate exercise capacity via an increased O_2_ extraction rather than changes in CO in T2DM patients [32]. Given the contradictory and inconsistent results, the role of exercise intensity in improving diastolic and systolic function in diet-induced T2DM models, likely with standardized exercise training protocols and diet composition, remains to be further elucidated. Although endurance exercise has been shown to have cardioprotective effects, it should be noted that some athletes performing prolonged physical exercise, including marathon running, show exercise-induced cardiac fatigue and have a higher risk of sudden cardiac arrest [88,89]. As such, amateur and trained marathon runners showed acutely increased plasma levels of cardiac injury markers N-terminal pro-brain natriuretic peptide and troponin I and T [90,91,92]. Furthermore, several studies reported a decrease in LV cardiac relaxation (i.e., lower e wave and ratio of peak mitral flow velocity in early versus late filling (e/a)) following marathon running or prolonged strenuous exercise in healthy, recreational runners [93,94]. De Bosscher et al. demonstrated that young endurance athletes can have a reduced EF, however, this is not associated with structural or functional abnormalities of the heart [95]. In the current study, no detrimental echocardiographic changes were observed after MIT and HIIT in WD-fed rats. This might indicate that the applied 12-week training period is too short to induce cardiac functional abnormalities or that our training protocol included appropriate recovery periods between the training interventions to induce physiological adaptation [96]. Nevertheless, future research should focus on the potential negative impact of prolonged exercise on cardiac function and how to adapt training protocols to avoid this, with special attention to exercise intensity.

### 4.3. Mechanisms Underlying the Curative Role of MIT and HIIT

Previously, we have shown that MIT and HIIT effectively improved cardiac function by preventing fibrosis and boosting oxidative metabolism in healthy rats [37]. In this study, we demonstrate that MIT and HIIT can similarly restore WD-induced T2DM metabolic and cardiac impairments. As such, both exercise training modalities significantly reduced the interstitial collagen deposition in the myocardium. These findings are in line with results reported by other studies, with some reporting attenuated cardiac fibrosis following 8 to 10 weeks of MIT but not HIIT in HFD-fed mice [78,97], while others confirming a reduced myocardial collagen content, assessed as hydroxyproline levels, after 10 weeks of HIIT [72]. Furthermore, mRNA expression of matrix metalloproteinase 2, an enzyme involved in cardiac extracellular matrix (ECM) degradation, has previously been shown to be decreased after both exercise training modalities [78,84,85]. Here, the exercise-induced decrease in fibrosis was accompanied by, even if not significant, clear lower protein levels of LOX following MIT. In silico predictive methods support that LOX-like 2, a LOX isoform known to play an important role in ECM cross-linking, can be used as an early biomarker for diabetic cardiomyopathy [98]. Contrary to our findings, 26 weeks of voluntary treadmill running by hypertensive rats induced increased gene and protein expression of LOX in the heart accompanied by diastolic dysfunction [99]. Given their longer training period, it remains to be investigated whether chronically sustained MIT and HIIT cause adverse adaptations in the heart of WD-induced T2DM rats. Altogether, the inhibition of myocardial fibrosis and LOX content are likely to explain the observed improvements in LV wall thickness and EDP after MIT and HIIT, possibly indicating their potential to limit myocardial wall stiffness.

In addition, we also investigated the effect of exercise training on plasma AGEs. AGEs are complex compounds formed by the irreversible glycation of amino acids, peptides or proteins [100]. A heat-treated diet high in sugar content can be an exogenous source of AGEs, whereas they also accumulate with aging in the blood and tissues, including the aorta and heart [101,102,103]. In the heart, AGEs exert detrimental effects by cross-linking ECM proteins in the presence or absence of LOX, thereby causing myocardial fibrosis and stiffening. In addition, they interact with the membrane receptor for AGEs (RAGE), inducing intracellular inflammation and oxidative stress, or with soluble RAGE (sRAGE), a systemic decoy ligand that protects against AGEs. In general, plasma and tissue AGE levels are known to be tightly linked to T2DM-associated cardiac dysfunction [102]. However, data on the effect of exercise training on AGE pathways is sparse and, if tested, explored in the plasma of healthy but aged individuals [104,105] or rodents [106,107]. In our study, we found that MIT, but not HIIT, lowers general plasma AGE levels in WD-induced T2DM rats. Accordingly, Boor et al. showed that MIT significantly lowers plasma AGEs, specifically Nε-carboxymethyllysine, in genetically-modified obese Zucker rats [108]. Additionally, two clinical studies demonstrated that MIT increases sRAGE levels along with improvement in cardiorespiratory fitness in individuals with T2DM [109,110]. Future work should focus on unraveling the effect of exercise training on the AGEs-RAGE pathway, not only in plasma but also in tissues, in T2DM individuals and diet-induced T2DM animal models. To align results with exogenous and endogenous AGE sources, dietary AGE measurements should be included, which we acknowledge to be a limitation of the current study [111].

The metabolic changes in T2DM, including hyperglycemia and lipotoxicity, can induce cardiomyocytes to release pro-inflammatory cytokines which may stimulate the development of myocardial fibrosis [112]. Luo et al. showed that silencing of the NOD-like receptor protein 3 (NLRP3) inflammasome led to decreased myocardial inflammation and LV fibrosis in an HFD-induced T2DM rat model, confirming the role of inflammation in the pathology [113]. In our study, we observed that sedentary, WD-fed rats had a higher protein content of TNF-α in LV tissue. MIT tended to reduce the TNF-α levels, demonstrating its anti-inflammatory potential. Previous studies also have shown decreased protein and gene expression of TNF-α and interleukin 1β (IL-1β) after 6 and 8 weeks of low-intensity swimming in HFD-fed rats and mice, respectively [114,115]. Furthermore, 20 weeks of MIT during an HFD regime inhibited the upregulation of NLRP3 and IL-1β, thereby deactivating cardiac inflammasome formation [77,116]. In our study, no changes in TNF-α levels in LV tissue were detected following HIIT. However, clinical studies demonstrate pro-inflammatory cytokine secretion after high-intensity exercise training, especially when performed without appropriate resting periods [117]. Additional research is needed to assess the either pro- or anti-inflammatory impact of the different exercise intensities. In particular, determining the type of immune cells involved might be of interest to better understand which exercise intensity is the most efficient regarding inflammation in diabetic cardiomyopathy.

Due to their key role in energy metabolism, mitochondria are important producers of ROS in cardiomyocytes [118]. In the diabetic heart, increased production of mitochondrial ROS and lower activity of anti-oxidant defense mechanisms have been suggested as contributing factors in the pathology [119,120]. Citrate synthase is the initial enzyme of the Krebs cycle and is important in the production of energy via mitochondrial respiration (i.e., oxidative phosphorylation) [121]. The enzyme is considered a biomarker for cellular oxidative capacity and mitochondrial content in striated muscle following a training regimen [37,122,123]. In this study, we observed an increased citrate synthase activity in LV tissue following MIT and HIIT. This result might indicate an improved oxidative adaptation and/or higher mitochondrial density following the training intervention, which remains to be directly confirmed. Wang et al. also identified that 16 weeks of MIT increased citrate synthase activity in isolated cardiac mitochondria of low-dose STZ treatment and HFD-induced T2DM mice, in line with our findings [124]. Furthermore, different studies describe improvements in mitochondrial structure, decreased ROS levels and elevated protein levels of antioxidant superoxide dismutase 2 in the heart after MIT [77,78,84,124] and HIIT [72,78] in HFD-induced T2DM models. Taken together, the current study cannot pinpoint one single mechanism but suggests the involvement of multiple pathways underlying the cardiac effect of exercise intensity in T2DM.

### 4.4. Limitations

The main limitation of the study is the presence of a large within-group variation in the sedentary WD group. Most likely, the large within-group variation of the WD-fed rats is attributable to the outbred nature of the Sprague–Dawley strain. Whereas their individual genetic makeup optimally recapitulates the clinical situation, it also implies a significant variability regarding the susceptibility to high-sugar or -fat diets regarding the progression of T2DM or obesity [125,126]. On the contrary, inbred stains (e.g., C57BL/6J mice) develop the disease more consistently in response to diets but show major differences in disease progression between males and females, which is not the case for Sprague–Dawley rats [18,65].

Current management of T2DM patients with CVD mainly includes pharmacological prescription, aiming at improved glycemic control while providing adjunct approaches to safeguard the heart [3,127]. Despite these interventions, many patients do not achieve the determined treatment goals [21]. Therefore, a multifactorial approach including exercise training prescription of MIT or HIIT should be considered. Several aspects should be taken into account when determining a tailored, individualized treatment. In this context, our animal model presents an important limitation, namely being performed in rats with uncontrolled diabetes and cardiac remodeling, whereas T2DM patients with cardiac comorbidities are often prescribed multiple drugs [48]. In addition, although the combination of MIT or HIIT and pharmacological interventions might work synergistically, appropriate research must rule out adverse, compensatory effects on cardiometabolic health [67]. Furthermore, training at high intensity can only be implemented as a treatment in T2DM patients under certain conditions. As such, HIIT should be avoided when disease-related barriers are present (i.e., neuropathy, retinopathy, neuropathy, myocardial ischemia and cardiac arrhythmia). Additionally, HIIT is also more demanding regarding the patient’s feasibility, as it requires a high level of motivation and supervision [25]. However, several clinical studies demonstrated that the adherence to HIIT was similar to MIT in cardiac rehabilitation patients [35]. Thus, future research should investigate the interaction between pharmacological interventions and exercise training intensities to identify the most optimal treatment, regarding efficacy, safety and feasibility, adjusted for each individual patient. demonstrates that both exercise intensities.

## 5. Conclusions

The current study shows that both MIT and HIIT are effective in alleviating T2DM with LV cardiac remodeling and dysfunction in a WD-induced rat model. Regarding metabolic influences, MIT had superior positive effects on insulin and plasma AGEs homeostasis whereas HIIT had a higher potential for lipid handling. In the heart, both MIT and HIIT treated diastolic dysfunction (i.e., decreased wall thickness and EDP), whereas only MIT normalized systolic function (i.e., increased EF and FS). The exercise-mediated cardiac adaptations were related to the reversal of LV cardiac remodeling and improved mitochondrial oxidative capacity by both MIT and HIIT, whereas only MIT lowered LV cardiac inflammation. As this study demonstrates that both exercise intensities are effective in improving structural and functional changes in the heart, it suggests a pivotal role for MIT as well as HIIT in the management of T2DM patients with cardiac comorbidity, adjusted to their feasibility.

## Figures and Tables

**Table 1:** Echocardiographic characteristics of rats after 18 weeks of Western diet.

	CD	WD
HR (beats/min)	354 ± 18	339 ± 8
AWT (mm)	1.49 ± 0.02	1.68 ± 0.03 ***
PWT (mm)	1.52 ± 0.03	1.82 ± 0.04 ****
EDD (mm)	7.17 ± 0.35	7.09 ± 0.18
ESD (mm)	4.11 ± 0.30	4.29 ± 0.15
EDV (µL)	325 ± 28	330 ± 18
ESV (µL)	86 ± 11	96 ± 7
SV (µL)	240 ± 20	234 ± 14
CO (mL/min)	86 ± 9	80 ± 5
EF (%)	74 ± 2	71 ± 1
FS (%)	43 ± 3	39 ± 2

Echocardiographic characteristics of rats receiving CD (n = 10) or W (n = 22) for 18 weeks. Data are shown as mean ± SEM. *** denotes *p* < 0.001 and **** denotes *p* < 0.0001 vs. CD. AWT, anterior wall thickness; CO, cardiac output; EF, ejection fraction; EDD, end-diastolic diameter; EDV, end-diastolic volume; ESD, end-systolic diameter; ESV, end-systolic volume; FS, fractional shortening; HR, heart rate; PWT, posterior wall thickness; SV, stroke volume.

**Table 2:** Effect of exercise training on echocardiographic and hemodynamic parameters of 30-week Western diet fed rats.

	CD	WD-SED	WD-MIT	WD-HIIT
HR (beats/min)	338 ± 12	334 ± 8	327 ± 19	320 ± 16
AWT (mm)	1.49 ± 0.02	1.84 ± 0.03 ****	1.63 ± 0.04	1.66 ± 0.05
PWT (mm)	1.61 ± 0.03	1.96 ± 0.03 ****	1.66 ± 0.05 ^##^	1.67 ± 0.08 ^##^
EDD (mm)	7.10 ± 0.29	7.60 ± 0.28	7.86 ± 0.44	7.54 ± 0.28
ESD (mm)	3.76 ± 0.24	4.91 ± 0.17 *	4.46 ± 0.36	4.66 ± 0.27
EDV (µL)	329 ± 27	392 ± 33	413 ± 41	371 ± 31
ESV (µL)	75 ± 10	136 ± 12 *	108 ± 18	110 ±10
SV (µL)	254 ± 20	255 ± 25	305 ± 26	261 ± 26
CO (mL/min)	83 ± 7	86 ± 10	99 ± 12	85 ± 11
EF (%)	78 ± 2	65 ± 2 **	75 ± 2 ^#^	70 ± 3
FS (%)	47 ± 2	35 ± 1 ***	44 ± 3 ^#^	38 ± 2
EDP (mmHg)	5 ± 1	14 ± 3 *	6 ± 1 ^#^	7 ± 1 ^#^
ESP (mmHg)	85 ± 2	99 ± 3 *	97 ± 3	100 ± 3
Tau (s)	0.013 ± 0.001	0.028 ± 0.015	0.015 ± 0.002	0.015 ± 0.001

Echocardiographic and hemodynamic characteristics at the end of the study of rats receiving a CD (n = 10) or either kept WD-SED (n = 8), undergoing WD-MIT (n = 7), or WD-HIIT (n = 7) for 12 weeks. Data represent mean ± SEM. * denotes *p* < 0.05, ** denotes *p* < 0.01, *** denotes *p* < 0.001 and **** denotes *p* < 0.0001 vs. CD. ^#^ denotes *p* < 0.05 and ^##^ denotes *p* < 0.01 vs. WD-SED. AWT, anterior wall thickness; CO, cardiac output; EF, ejection fraction; EDD, end-diastolic diameter; EDP, end-diastolic pressure; EDV, end-diastolic volume; ESD, end-systolic diameter; ESP, end-systolic pressure; ESV, end-systolic volume; FS, fractional shortening; HR, heart rate; PWT, posterior wall thickness; SV, stroke volume; Tau, time constant for isovolumetric relaxation.

## Data Availability

The data presented in this study are available on request from the corresponding author.

## Abbreviations

AGEs	Advanced glycation end-products
AWT	Anterior wall thickness
CD	Standard chow diet
CO	Cardiac output
CVD	Cardiovascular diseases
e/a	Ratio of peak mitral flow velocity in early versus late filling
e’	Peak septal mitral annulus velocity in early filling phase
e/e’	Ratio of peak mitral flow velocity versus peak mitral annular velocity
ECL	Enhanced chemiluminescence
ECM	Extracellular matrix
EDD	End-diastolic diameter
EDP	End-diastolic pressure
EDV	End-diastolic volume
EF	Ejection fraction
ESC	European Society for Cardiology
ESD	End-systolic diameter
ESP	End-systolic pressure
ESV	End-diastolic volume
FFA	Free fatty acids
FS	Fractional shortening
HbA1c	Blood hemoglobin A1c
HF	Heart failure
HFD	High fat diet
HFpEF	Heart failure with preserved ejection fraction
HFrEF	Heart failure with reduced ejection fraction
HIIT	High-intensity interval training
HOMA-IR	Homeostatic model assessment for insulin resistance
HR	Heart rate
IL-1β	Interleukin 1 beta
i.p.	Intraperitoneally
LOX	Lysyl oxidase
LV	Left ventricular
NLRP3	NOD-like receptor protein 3
MIT	Moderate-intensity training
OGTT	Oral glucose tolerance test
PWT	Posterior wall thickness
RAGE	Receptor for advanced glycation end-products
ROS	Reactive oxygen species
SED	Sedentary lifestyle
SEM	Standard error of the mean
sRAGE	Soluble receptor for advanced glycation end-products
STZ	Streptozotocin
SV	Stroke volume
T2DM	Type 2 diabetes mellitus
Tau	Time constant for isovolumetric relaxation
TBS-T	Tris-buffered solution containing 0.1% Tween-20
TL	Tibia bone length
TNF-α	Tumor necrosis factor alpha
WD	Western diet
